# Maternal and neonatal outcomes of pregnancies of infertile women during the COVID-19 pandemic: a real world evidence

**DOI:** 10.5935/1518-0557.20210119

**Published:** 2022

**Authors:** Batool Hossein Rashidi, Fatemeh Bandarian, Mahin Bandarian

**Affiliations:** 1 Health Reproductive Research Center, Imam Khomeini complex Hospital, Tehran University of Medical Sciences, Tehran, Iran; 2 Metabolomics and Genomics Research Center, Endocrinology and Metabolism Molecular- Cellular Sciences Institute, Tehran University of Medical Sciences, Tehran, Iran; 3 Endocrinology and Metabolism Research Center, Endocrinology and Metabolism Clinical Sciences Institute, Tehran University of Medical Sciences, Tehran, Iran; 4 Dep. of Obstetrics and Gynecology, Ziaeian Hospital, Tehran University of Medical Sciences, Tehran, Iran

**Keywords:** COVID-19 pandemic, pregnancy, outcome, IVF, ART

## Abstract

**Objective:**

The COVID-19 pandemic began in Dec. 2019 and its effects on pregnancy outcomes are still unknown. This study aimed to evaluate the pregnancy outcomes of infertile women who conceived during the COVID-19 pandemic.

**Methods:**

This cross-sectional study included infertile women who conceived during the COVID-19 pandemic. Infertile women referred to the infertility center at the Vali-e-Asr hospital who conceived spontaneously or with the aid of ART (IUI, ICSI) were included and followed until delivery or pregnancy termination.

**Results:**

A total of 38 pregnant women (34 conceiving after ART and four spontaneously) were included. Seventeen (44.74%) of the 38 pregnant women developed COVID-19 symptoms. No significant difference was detected in maternal and neonatal outcomes, including miscarriage, PROM, low birth weight, or premature birth between pregnancies with and without COVID-19 symptoms. A significant difference was found between the two groups in delivery route.

**Conclusions:**

No associations were found with maternal and neonatal morbidity in women conceiving during the COVID-19 pandemic and in pregnant women with and without COVID-19 symptoms.

## INTRODUCTION

Coronavirus Disease 2019 (COVID-19) is an emergent widely disseminated condition (Yang *et al*., 2020). After reports of increasing numbers of individuals with COVID-19 and deaths within a short period of time in numerous countries, the WHO declared COVID-19 a pandemic on March 11, 2020 (Monteleone *et al*., 2020). COVID-19 is a fast transmitting infection (The Novel Coronavirus Pneumonia Emergency Response Epidemiology Team, 2020) that presents with symptoms ranging from common cold to severe acute respiratory distress syndrome (Wang *et al*., 2020a)

A critical measure taken to manage any communicable disease is providing care to susceptible persons (Alfaraj *et al*., 2019; Wong *et al*., 2004). Cardiorespiratory and immune changes in pregnancy increase susceptibility of this population to severe infection and hypoxia (Guan *et al*., 2020), in addition to changing the hormonal milieu, especially in early pregnancy as a result of adaptive changes to fetal antigens, and influencing immune response to viral infection, an important element due to fetal organ development (Littauer *et al*., 2017; Wong *et al*., 2004).

Recent data showed that pregnant women diagnosed with COVID-19 have similar symptoms and clinical characteristics than their non-pregnant counterparts; no cases of vertical transmission in late pregnancy have been confirmed (Monteleone *et al*., 2020). There is no data available today on COVID-19 and obstetric complication during the first trimester of pregnancy (Monteleone *et al*., 2020). Fetal complications from COVID-19 include miscarriage (2%), IUGR (10%), and preterm birth (39%). There is no data on perinatal outcomes when infection strikes during early pregnancy (Chen *et al*., 2020a).

The severe acute respiratory syndrome coronavirus 2 (SARS-CoV-2) uses ACE2 as a cell receptor and expression of RNA; ACE2 levels in the placenta between weeks 6 and 14 of pregnancy are low, therefore transmission of SARS-CoV-2 from the mother to the fetus in the first trimester seem unlikely, although severe maternal respiratory failure and disturbed placenta flow may cause miscarriage (Lamouroux *et al*., 2020). More definitive evidence is needed to counsel mothers about the risk of congenital anomalies from SARS-CoV-2 infection (Lamouroux *et al*., 2020).

This study aimed to evaluate pregnancy and neonatal outcomes and morbidity in infertile women who conceived during the COVID-19 pandemic, spontaneously or with the aid of ART, to identify potential impacts of COVID-19 on pregnancy, especially in the first trimester.

## MATERIALS AND METHODS

This cross-sectional study included infertile women who conceived during the COVID-19 pandemic. Women with a history of infertility referred to the Vali-e-Asr hospital infertility center for treatment, who conceived spontaneously or by ART (Intrauterine insemination (IUI), Intracytoplasmic sperm injection (ICSI)), were included in this study after confirmation of clinical pregnancy by observation of a gestational sac in ultrasound examination. Patients with chemical pregnancies (positive ß-hcG) without a gestational sac or fetal heart beats in ultrasound examination were excluded from the study.

All patients signed informed consent terms before enrollment and the Ethics Committee at the Vali-e-Asr Reproductive Health Research center approved the study protocol. Pregnancy and neonatal outcomes and morbidity of eligible women were evaluated via questionnaires and telephone interviews. Patients were also asked about the presence of COVID-19 symptoms during pregnancy.

Primary endpoints included pregnancy morbidities such as preeclampsia, IUGR (intrauterine growth retardation), PTL (preterm labor), miscarriage, and PROM (premature rupture of membranes), as well as fetal anomalies in patients with and without COVID-19 symptoms during pregnancy. Secondary endpoints were gestational diabetes, neonatal morbidity, and postpartum complications, which were compared between women with and without COVID-19 symptoms.

## RESULTS

This cross sectional study included 38 pregnant women. Thirty-four women (89.5%) conceived with the aid of ART and four (10.5%) conceived spontaneously. In the group of women who conceived with the aid of ART, 14.70% (5/34) conceived through IUI and 85.30% (29/34) with ICSI. Table 1 shows the characteristics and outcomes of the pregnancies of the included women seen during the COVID-19 pandemic. Four (10.5%) were twin pregnancies. Miscarriage occurred in five pregnancies, all in the ICSI group (5/34, 14.7%) and none with COVID-19 symptoms. One ectopic pregnancy (1/5; 20%) occurred in a patient without COVID-19 symptoms submitted to IUI. Neonates with low birth weight (fetal weight < 2500 g) were seen in eight pregnancies (23.5%; 8/34), seven from ICSI (20.6%) and one from IUI (Table 1). Nine patients (23.6%) required hospitalization, one (11.1%) for COVID-19 symptoms, two (22.2%) for gestational diabetes, one for hypertension (11.1%), and one for gestational diabetes and hypertension (11.1%).

**Table 1:** Characteristics and outcomes of pregnancies of infertile women during the COVID-19 pandemic.

	Spontaneous n=4	ART		
		IUI n=5	ICSI n=29	Total ARTs n=34
**Age (mean± SD), Year**	31.67±7.37	36.6±7.7	30.54±6.06	31.45±6.58
**BMI (mean± SD), Kg/m^2^**	28.56±3.02	26.15±6.17	26.68±3.97	26.61±4.19
**Twin, n (%)** **Singleton, n (%)**	0 (0) 4/4 (100)	0 (0) 4/5 (80)	4/29 (13.8) 20/29 (68.96)	4/34 (11.76) 24/34 (70.6)
**Ectopic Pregnancy**	0 (0)	1/5 (20)	0 (0)	1/34 (2.94)
**Miscarriage**	0 (0)	0 (0)	5/29 (17.24)	5/34 (14.7)
**Diabetes, n (%)**	3/4 (75)	2/5 (40)	5/29 (17.24)	7/34 (20.6)
**HTN, n (%)**	1/4 (25)	1/5 (20)	4/29 (13.8)	5/34 (14.7)
**PROM, n (%)**	-	-	2/29 (6.9)	2/34 (5.9)
**Birth Weight, n (%)** **<1500 gr** **<2500 gr** **>4000 gr**	0/4 (0) 0 (0) 0 (0)	0/5 (0) 1/5 (20) 1/5 (20)	1/29 (3.44) 7/29 (24.13) 1/29 (3.44)	1/34 (2.94) 8/34 (23.52) 2/34 (5.9)
**Hospital Admission during pregnancy, n (%)**	1/4 (25)	0/5 (0)	8/29 (27.6)	8/34 (23.52)
**Postpartum complication Bleeding**	0 (0)	0 (0)	1/29 (3.44)	1/34 (2.94)
**Neonate gender, n (%)** **Male Female**	2/4 (50) 2/4 (50)	- 4/5 (80)	14/29 (48.3) 14/29 (48.3)	14/34 (41.2) 18/34 (52.94)
**Prematurity, n (%)** **GA at birth<32w** **GA at birth<37w**	- -	- 1/5 (20)	1/29 (3.4) 6/29 (20.7)	1/34 (2.9) 7/34 (20.6)
**Neonatal hospital admission, n (%)** **Ward admission** **NICU admission**	- 1/4 (25)	1/5 (20) 1/5 (20)	5/29 (17.24) 8/29 (27.6)	6/34 (17.64) 9/34 (26.5)
**Neonatal RDS**	-	-	3/29 (10.34)	3/34 (8.82)
**Neonatal anomaly, n (%)**	0/4 (0)	2/5 (40)	2/29 (6.9)	4/34 (11.76)
**Anomaly type, n (%)** **CDH** **Skin Hemangioma** **Club foot** **Imperforated anus** **Hydronephrosis**	- - - - -	1/5 (20) 1/5 (20) - - -	- - 1/29 (3.4) 1/29 (3.4) -	

The route of delivery was a Cesarean section (C-section) in 73.7% of the cases (28/38) and normal vaginal delivery (NVD) in 10.5% (4/38) of the cases. Morbidity during pregnancy was seen in 15/38 (39.5%) of the cases. Gestational diabetes was the most common morbidity with 26.3% (10/38) of the cases, followed by hypertension with 15.8% (6/38) and PROM with 5.26% (2/38), respectively (Table 1). Postpartum complications including hemorrhage and hysterectomy were seen in one case (2.63%).

Seventeen of 38 pregnant women (44.74%) experienced COVID-19 symptoms (respiratory or gastrointestinal), 47.1% in the first trimester (8/17); 29.4% in the second trimester (5/17); 23.5% (4/17) in third trimester; and 11.8% (2/17) in the first week postpartum. However, two women experienced COVID-19 symptoms twice during pregnancy; one during the first and third trimester of pregnancy and one during the first trimester of pregnancy and postpartum. Three of fifteen mothers (20%) tested positive for COVID-19 in PCR tests performed for any reason. None of the patients included in the study developed severe COVID-19.

Regarding neonatal outcomes, the mean gestational age at birth was 37.41±1.91 weeks and the mean neonatal birth weight was 2945.83±868.48 g. Sixteen neonates (44.4%; 16/36) were males and 20 (55.5%) were females. Neonatal anomalies were seen in 10.5% (4/38) of all pregnancies; 15.8% (6/38) of the neonates were admitted to the neonatal ward and 26.3% (10/38) to the NICU for different reasons. RDS was seen in 10.3% (3/29) of the neonates in the ICSI group, and in none of the IUI and spontaneous conception groups. Regarding neonatal complications, very low birth weight (fetal weight < 1500 g) was observed in one pregnancy (1/38, 2.63%); low birth weight (fetal weight < 2500 g) in 21.1% (8/38); premature birth at gestational age < 37 weeks in 7/38 (18.42%); and gestational age < 32 weeks in 1/38 (2.63%) of the pregnancies.

PCR testing for COVID-19 was performed in two neonates due to screening and all were negative. None of the neonates presented COVID-19 symptoms, although four mothers experienced COVID-19 symptoms in the third trimester, five in the second trimester, eight in the first trimester, and two in the first week postpartum.

There was no significant difference in maternal and neonatal outcomes, including miscarriage, PROM, low birth weight, and premature birth, between pregnancies with and without COVID-19 symptoms. A significant difference was found between the two groups in delivery route, in that all pregnant women with COVID-19 symptoms had C-sections (*p*: 0.03).

Comparison of singleton and twin pregnancies found that women with twin pregnancies had significantly more deliveries at gestational ages of less than 37 weeks (75% in twin *vs*. 16.7% in singleton pregnancies, *p*=0.04). PROM was also significantly more frequent in twin than in singleton pregnancies (50% in twin *vs*. 0% in singleton pregnancies, *p*=0.02). No significant differences in delivery route or other maternal and neonatal complications including postpartum complications, diabetes, hypertension, as well as neonatal low birth weight, neonatal hospital admission, and anomalies were seen between singleton and twin pregnancies (*p*>0.05).

The comparison of pregnancy outcomes and maternal and fetal complications between IUI, ICSI, and spontaneous pregnancies showed that although all miscarriages occurred in ICSI pregnancies (5/34, 14.7%), the difference between groups was not significant (*p*=0.45). C-section was the delivery route in 91.7% of ICSI pregnancies, 14.3% of IUI pregnancies, and 50% of spontaneous pregnancies; the difference was statistically significant (*p*=0.047). Neonatal anomalies were more common in IUI pregnancies (40%) compared to ICSI (6.9%) and spontaneous pregnancies (0%); the difference was not significant by a thin margin (*p*=0.064). There was no significant difference in other maternal or neonatal outcomes and complications between the three groups of pregnancies (IUI, ICSI, and spontaneous) (*p*>0.05).

## DISCUSSION

All five miscarriages reported in this study involved women submitted to ICSI without COVID-19 symptoms. Therefore, no significant relationship was identified between COVID-19 and abortion. One study reported a 2.5% spontaneous abortion rate among mothers with positive COVID-19 tests (Patanè *et al*., 2020). One meta-analysis reported two miscarriages in 324 pregnant women with COVID-19, but the study in question had no control group (Baud *et al*., 2020). Isolated case reports have suggested that late miscarriage may be a manifestation of COVID-19 (Baud *et al*., 2020; Hachem *et al*., 2020). We had a late miscarriage at 20 weeks of gestation, but the patient did not have COVID-19 symptoms. Prevalence of miscarriage in our study was similar or lower than other studies enrolling ICSI populations. Miscarriage rates of 16% and 14% have been reported in ICSI pregnancies (Ashrafi *et al*., 2014). Bahceci & Ulug (2005) reported a rate of 20% for first-trimester pregnancy losses.

Some case reports found increasing stillbirth rates in pregnant women tested positive for COVID-19 (Moore *et al*., 2016; Wang *et al*., 2020b). Maternal critical illness has been associated with fetal demise (Dotters-Katz & Hughes, 2020). An early report demonstrated concerns about stillbirth in patients with COVID-19 confirmed patients - 6.9 *vs*. 1.19 per 1000 births (Khalil *et al*., 2020). In the present study, no stillbirths or neonatal deaths were observed. This may be due to the lack of critically ill patients in our study. Only one woman was hospitalized for COVID-19. She did not have severe disease and experienced mild liver enzyme increases. Her baby was born on week 37 of gestation. Our results are in line with a study that showed no fetal or neonatal deaths, but found a 50% incidence of preterm labor. However, the study in question did not differentiate between iatrogenic or spontaneous preterm labor (Ciobanu *et al*., 2020).

Another study found no difference in preeclampsia, gestational diabetes, or PROM between patients with and without COVID-19 (Yang *et al*., 2020). The authors found that COVID-19 did not increase the risk of preeclampsia or PROM (Yang *et al*., 2020). The impact of COVID-19 in preterm births has not been specifically described and the occurrence of IUGR in mothers with COVID-19 is unclear (Ciobanu *et al*., 2020). Evidence for preterm birth is not well documented (Dotters-Katz & Hughes, 2020). Similarly, our study showed no significant difference in maternal and neonatal outcomes between pregnancies with and without COVID-19 symptoms. However, the only significant difference between the two groups was in delivery route, in that all women with COVID-19 symptoms had C-sections.

Only one singleton preterm birth (< 34 weeks), involving a patient submitted to ICSI, was observed in our study, as described in other studies (Cavoretto *et al*., 2018). Similarly to another study (Zhu *et al*., 2016), three babies (8.8%) from singleton pregnancies had low birth weight (neonatal weight < 2500 g). A comparison between patients with and without COVID-19 symptoms revealed no significant differences in maternal and neonatal outcomes in the present study.

A case-control study found no significant differences in fetal and neonatal outcomes (fetal asphyxia, preterm birth, and fetal distress) between the two groups (Yang *et al*., 2020), as also seen in our study. In the present study, neonatal anomalies were detected in 10.5% of the newborns (2/5, 40% in the IUI and 2/29, 6.9% in the ICSI group), while in another study the prevalence of neonatal anomalies in women submitted to IVF, ICSI, and natural conception were 7.1%, 9.9%, and 5.7%, respectively (Davies *et al*., 2017). Prevalence of neonatal anomalies in mothers with and without COVID-19 symptoms was similar and no significant correlation was found between COVID-19 symptoms and neonatal anomalies. Nevertheless, the prevalence of neonatal anomalies was high in our study, possibly due the presence of COVID symptoms seen in 3 of 4 mothers with newborns with neonatal anomalies, two of which in the first trimester. This may suggest a correlation between COVID-19 and fetal anomaly. However, this assumption requires confirmation of infection through positive COVID-19 test results for all mothers of neonates with anomalies. In our study, only one of the three mothers in this group tested positive for COVID-19.

None of our neonates had COVID-19 symptoms or positive tests for the disease within 48 hours of birth, despite the presence of COVID-19 symptoms in four mothers in the third trimester and early postpartum. This result is in line with other studies that rejected vertical transmission of COVID-19 (Chen *et al*., 2020a;b; Karimi-Zarchi *et al*., 2020; Zhu *et al*., 2020). Prevalence of postpartum complication is shown in Table 1. The comparison of morbidities found no difference between patients with or without COVID-19 symptoms. In one study, the prevalence of postpartum complications such as postpartum hemorrhage in ICSI patients was 8.3% *versus* 2.9% in patients conceiving normally; the difference between singleton ART pregnancy and controls was 4.9% *vs*. 2.9%, and between twin ART pregnancy and controls was 11.5% *vs*. 4.5% (Zhu *et al*., 2016). In the present study, with the exception of delivery route, we found no significant differences in maternal and neonatal outcomes of pregnancy between the three study groups - IUI, ICSI, and spontaneous conception.

This study is the first to report the pregnancy outcomes of infertile women conceiving during the COVID-19 pandemic. As such, it might provide preliminary data about the impact of COVID-19 in early pregnancy and in the first trimester of pregnancy. The small sample size and the lack of confirmed COVID-19 test results for most of the women with COVID-19 symptoms were two limitations of the current study.

This study found no correlation between maternal and neonatal morbidity during pregnancy and in postpartum in women conceiving during the COVID-19 pandemic and in pregnant women with and without COVID-19 symptoms. Our results did not support the existence of vertical transmission of COVID-19. A higher rate of neonatal anomalies was observed in our study, thereby indicating a potential correlation between COVID-19 and neonatal anomalies; however, additional evidence is needed to confirm such correlation.

## References

[r1] Alfaraj SH, Al-Tawfiq JA, Memish ZA (2019). Middle East Respiratory Syndrome Coronavirus (MERS-CoV) infection
during pregnancy: Report of two cases & review of the
literature. J Microbiol Immunol Infect.

[r2] Ashrafi M, Gosili R, Hosseini R, Arabipoor A, Ahmadi J, Chehrazi M (2014). Risk of gestational diabetes mellitus in patients undergoing
assisted reproductive techniques. Eur J Obstet Gynecol Reprod Biol.

[r3] Bahceci M, Ulug U (2005). Does underlying infertility aetiology impact on first trimester
miscarriage rate following ICSI? A preliminary report from 1244 singleton
gestations. Hum Reprod.

[r4] Baud D, Greub G, Favre G, Gengler C, Jaton K, Dubruc E, Pomar L (2020). Second-Trimester Miscarriage in a Pregnant Woman With SARS-CoV-2
Infection. JAMA.

[r5] Cavoretto P, Candiani M, Giorgione V, Inversetti A, Abu-Saba MM, Tiberio F, Sigismondi C, Farina A (2018). Risk of spontaneous preterm birth in singleton pregnancies
conceived after IVF/ICSI treatment: meta-analysis of cohort
studies. Ultrasound Obstet Gynecol.

[r6] Chen H, Guo J, Wang C, Luo F, Yu X, Zhang W, Li J, Zhao D, Xu D, Gong Q, Liao J, Yang H, Hou W, Zhang Y (2020a). Clinical characteristics and intrauterine vertical transmission
potential of COVID-19 infection in nine pregnant women: a retrospective
review of medical records. Lancet.

[r7] Chen S, Huang B, Luo DJ, Li X, Yang F, Zhao Y, Nie X, Huang BX (2020b). Pregnancy with new coronavirus infection: clinical
characteristics and placental pathological analysis of three
cases. Zhonghua Bing Li Xue Za Zhi.

[r8] Ciobanu AM, Peltecu G, Panaitescu AM (2020). Coronavirus in pregnancy. What we know so far? Maedica.

[r9] Davies MJ, Rumbold AR, Marino JL, Willson K, Giles LC, Whitrow MJ, Scheil W, Moran LJ, Thompson JG, Lane M, Moore VM (2017). Maternal factors and the risk of birth defects after IVF and
ICSI: a whole of population cohort study. BJOG.

[r10] Dotters-Katz SK, Hughes BL (2020). Considerations for Obstetric Care during the COVID-19
Pandemic. Am J Perinatol.

[r11] Guan WJ, Ni ZY, Hu Y, Liang WH, Ou CQ, He JX, Liu L, Shan H, Lei CL, Hui DSC, Du B, Li LJ, Zeng G, Yuen KY, Chen RC, Tang CL, Wang T, Chen PY, Xiang J, Li SY (2020). China Medical Treatment Expert Group for Covid-19. Clinical
Characteristics of Coronavirus Disease 2019 in China. N Engl J Med.

[r12] Hachem R, Markou GA, Veluppillai C, Poncelet C (2020). Late miscarriage as a presenting manifestation of
COVID-19. Eur J Obstet Gynecol Reprod Biol.

[r13] Karimi-Zarchi M, Neamatzadeh H, Dastgheib SA, Abbasi H, Mirjalili SR, Behforouz A, Ferdosian F, Bahrami R (2020). Vertical Transmission of Coronavirus Disease 19 (COVID-19) from
Infected Pregnant Mothers to Neonates: A Review. Fetal Pediatr Pathol.

[r14] Khalil A, von Dadelszen P, Draycott T, Ugwumadu A, O’Brien P, Magee L (2020). Change in the Incidence of Stillbirth and Preterm Delivery During
the COVID-19 Pandemic. JAMA.

[r15] Lamouroux A, Attie-Bitach T, Martinovic J, Leruez-Ville M, Ville Y (2020). Evidence for and against vertical transmission for severe acute
respiratory syndrome coronavirus 2. Am J Obstet Gynecol.

[r16] Littauer EQ, Esser ES, Antao OQ, Vassilieva EV, Compans RW, Skountzou I (2017). H1N1 influenza virus infection results in adverse pregnancy
outcomes by disrupting tissue-specific hormonal regulation. PLoS Pathog.

[r17] Monteleone PA, Nakano M, Lazar V, Gomes AP, Martin de H, Bonetti TC (2020). A review of initial data on pregnancy during the COVID-19
outbreak: implications for assisted reproductive treatments. JBRA Assist Reprod.

[r18] Moore SA, Dietl CA, Coleman DM (2016). Extracorporeal life support during pregnancy. J Thorac Cardiovasc Surg.

[r19] Patanè L, Morotti D, Giunta MR, Sigismondi C, Piccoli MG, Frigerio L, Mangili G, Arosio M, Cornolti G (2020). Vertical transmission of coronavirus disease 2019: severe acute
respiratory syndrome coronavirus 2 RNA on the fetal side of the placenta in
pregnancies with coronavirus disease 2019-positive mothers and neonates at
birth. Am J Obstet Gynecol MFM.

[r20] The Novel Coronavirus Pneumonia Emergency Response Epidemiology
Team (2020). The Epidemiological Characteristics of an Outbreak of 2019 Novel
Coronavirus Diseases (COVID-19) - China, 2020. China CDC Wkly.

[r21] Wang D, Hu B, Hu C, Zhu F, Liu X, Zhang J, Wang B, Xiang H, Cheng Z, Xiong Y, Zhao Y, Li Y, Wang X, Peng Z (2020a). Clinical Characteristics of 138 Hospitalized Patients With 2019
Novel Coronavirus-Infected Pneumonia in Wuhan, China. JAMA.

[r22] Wang X, Zhou Z, Zhang J, Zhu F, Tang Y, Shen X (2020b). A Case of 2019 Novel Coronavirus in a Pregnant Woman With Preterm
Delivery. Clin Infect Dis.

[r23] Wong SF, Chow KM, Leung TN, Ng WF, Ng TK, Shek CC, Ng PC, Lam PW, Ho LC, To WW, Lai ST, Yan WW, Tan PY (2004). Pregnancy and perinatal outcomes of women with severe acute
respiratory syndrome. Am J Obstet Gynecol.

[r24] Yang Z, Wang M, Zhu Z, Liu Y (2020). Coronavirus disease 2019 (COVID-19) and pregnancy: a systematic
review. J Matern Fetal Neonatal Med.

[r25] Zhu H, Wang L, Fang C, Peng S, Zhang L, Chang G, Xia S, Zhou W (2020). Clinical analysis of 10 neonates born to mothers with 2019-nCoV
pneumonia. Transl Pediatr.

[r26] Zhu L, Zhang Y, Liu Y, Zhang R, Wu Y, Huang Y, Liu F, Li M, Sun S, Xing L, Zhu Y, Chen Y, Xu L, Zhou L, Huang H, Zhang D (2016). Maternal and Live-birth Outcomes of Pregnancies following
Assisted Reproductive Technology: A Retrospective Cohort
Study. Sci Rep.

